# Carvacrol and rosemary oil at higher concentrations induce apoptosis in human hepatoma HepG2 cells

**DOI:** 10.2478/intox-2014-0027

**Published:** 2015-03-04

**Authors:** Martina Melušová, Soňa Jantová, Eva Horváthová

**Affiliations:** 1 Institute of Biochemistry, Nutrition and Health Protection, Faculty of Chemical and Food Technology, Slovak University of Technology, Bratislava, Slovakia; 2 Department of Genetics, Cancer Research Institute, Slovak Academy of Sciences, Bratislava, Slovakia

**Keywords:** carvacrol, rosemary oil, apoptosis, HepG2 cells

## Abstract

Natural essential oils are volatile herbal complex compounds which manifest cytotoxic effects on living cells depending on their type and concentration but usually they are not genotoxic. Our previous studies showed that carvacrol (CA) and rosemary essential oil (RO) induced growth inhibition of both human cell lines HepG2 and BHNF-1, with hepatoma HepG2 cells being more sensitive to either compound tested. Cytotoxic concentrations of CA and RO induced the formation of DNA strand breaks. Further *ex vivo* studies showed that extracts prepared from hepatocytes of CA- and RO-supplemented rats did not increase incision repair activity compared to extracts from liver cells of control animals. Therefore, the aim of this work was to determine the effect of cytotoxic concentrations of CA and RO on the cell cycle and the ability of both natural volatiles to induce DNA fragmentation and apoptotic death of human hepatoma HepG2 cells. These effects were measured after 24 h incubation of HepG2 cells with CA and RO using three independent methods – flow cytometry, internucleosomal DNA fragmentation (electrophoresis) and micronucleus assay. Evaluation of morphological changes and formation of micronuclei in HepG2 cells showed no increase in the number of micronuclei in cells treated by CA and RO compared to control cells. On the other hand, CA and RO induced morphological changes typical for apoptosis in concentration-dependent manner. The presence of necrosis was negligible. Both natural compounds caused shrinking of cytoplasmic membrane and formation of apoptotic bodies. In addition, the highest concentrations of CA and RO induced internucleosomal DNA fragmentation (formation of DNA ladder) in HepG2 cells. Cell cycle analysis revealed the accumulation of cells in the G1 phase, which was accompanied by a reduction in the number of cells in the S phase after 24 h exposure to the substances tested. The cell division was thus slowed down or stopped and this process resulted in cell death.

## Introduction

Plant products and their derivatives have historically been invaluable as a source of therapeutic agents. However, in the past decade, research that aims at natural products in the pharmaceutical industry has declined (Koehn & Carter, 2005). Natural compounds have taken a secondary role in drug discovery and drug development after the advent of molecular biology and combinatorial chemistry made possible the rational design of chemical compounds to target specific molecules. Over the past few years, however, we have seen a renewed interest in the use of phytochemicals and, more importantly, their role as a basis for drug development (Ji *et al.*, 2009). Flowers, berries, leaves, barks and roots of different plants have been used through the ages as a source of flavor in food and perfume preparations. The volatiles responsible for the flavor of botanicals can be extracted from the plant material as “essential oils”, called also volatile oils. The term essential is intended to indicate that the oil is the fragrant essence of the plant from which it is extracted. Essential oils are constituted by hydrocarbons (monoterpenes and sesquiterpenes) and oxygenated compounds (alcohols, esters, ethers, aldehydes, ketones, lactones, phenols and phenol ethers). Plant volatiles are common components of the human diet. Therefore the increased human exposure to such products (a result of their application as crop protectants, food additives, *etc*.) requires a careful re-assessment of their toxicity and genotoxicity on the level of mammalian cells (Slamenova & Horvathova, 2013a).

Carvacrol (5-isopropyl-2-methyl phenol) is a constituent of the essential oil isolated from *Origanum hirtum*, oil obtained from pepperwort, wild bergamot and several other essential oils. It can be also prepared synthetically. Carvacrol minimizes oxidation of the lipid components in foods and can serve as a natural replacement of synthetic antioxidative food additives (Burt, 2004).

*Rosmarinus officina*lis L. is the most used aromatic and medicinal plant worldwide due to its essential oil, content of phenolic compounds and bioactive properties (Rozman & Jersek, 2009). The rosemary plant and its constituents are increasingly studied because of their positive effects on human health. Rosemary essential oil has already been used as a preservative in food industry due to its antioxidant and antimicrobial activities, yet it was shown to possess additional health benefits. Raskovic *et al.* (2014) demonstrated that rosemary essential oil, besides exhibiting free radical scavenging activity determined by DPPH assay, mediated its hepatoprotective effects also through activation of physiological defense mechanisms.

In the present study we used different concentrations of natural volatiles carvacrol (CA) and rosemary oil (RO) to observe changes in the cell cycle as well as the occurrence of apoptosis in the human hepatoma HepG2 cell line after 24 h treatment. Based on obtained results, we conclude that both natural volatiles induced apoptosis at cytotoxic concentrations. Our data also indicate that consumption of the natural compounds tested, CA and RO in the diet, might be valuable in the prevention of lifestyle diseases.

## Material and methods

### Plant volatiles

The plant volatiles examined in this study were: carvacrol (CA; Fluka, Buchs, Switzerland, purum ≥97%; density=0.974 g/ml; Mw=150.22) and *R. officinalis* essential oil (RO; Calendula Inc., Nová Ľubovňa, Slovakia, lot 5-014-009-12-06, containing approximately 25% 1,8-cineole, 19% α-pinene, 19% camphor, 17% *p*-cymene, 9% camphene, 5% β-pinene, 2% borneol and 4% of unidentified components as specified by manufacturer). Both volatiles were kept at room temperature. CA was diluted to concentrations of 50–650 μM in complete RPMI 1640 medium immediately before use. RO was dissolved in Cremophor EL^®^ (CrEL; Fluka, Sigma-Aldrich Co., Steinheim, Germany) and 70 °C serum-free RPMI 1640 medium to 1.25% stock solution which was diluted to final concentrations of 12.5–125×10^–3^‰ in complete RPMI 1640 medium. The final concentrations of Cremophor EL in the medium never exceeded 0.1% (in either control or treated cells), which did not affect cell viability.

### Chemicals

Chemicals were purchased from the following sources: Proteinase K and RNA-se from Biocom (Bratislava, Slovakia); agarose, ethidium bromide (EtBr), ethylenediaminetetraacetic acid (EDTA) and Tris(hydroxymethyl)aminomethane (Tris) from Sigma (St. Louis, USA); benzo[a]pyrene (B[a]P), DAPI (4‘,6-diamidino-2-phenylindole), DMSO, propidium iodide (PI) and Triton X-100 from Sigma (Sigma-Aldrich Co., Steinheim, Germany); fetal calf serum (FCS), kanamycin, penicillin/streptomycin and Roswell Park Memorial Institute (RPMI) 1640 medium from Invitrogen™ Gibco^®^ Life technologies Ltd., UK; phosphate-buffered saline (PBS; Mg^2+^- and Ca^2+^-free) from OXOID LIMITED (Basingstoke, UK); Ultra Safe Blue was obtained from Syngene, UK. Other chemicals were of analytical grade from commercial suppliers.

### Cell culture

Human hepatoma HepG2 cell line was obtained from Prof. A.R. Collins (University of Oslo, Oslo, Norway). Cells were cultured in RPMI 1640 medium supplemented with 10% fetal calf serum and antibiotics (penicillin 200 U/ml/streptomycin 100 μg/ml and kanamycin 100 μg/ml) on plastic Petri dishes at 37 °C in humidified atmosphere of 5% CO_2_.

### Treatment of cells

Exponentially growing HepG2 cells were treated with different concentrations of CA or RO for 24 h on Petri dishes.

The stock solution of B[a]P (2 mM in DMSO) was diluted in complete RPMI medium immediately before use to a final concentration of 0.5 μM. After treatment with B[a]P (1 h), cells were irradiated with UVA light (2.4 J/cm^2^) and used as positive control in electrophoretic analysis of apoptosis.

### Micronucleus assay

The signs of apoptosis and the number of micronuclei after 24 h exposure of HepG2 cells to individual concentrations of CA (300–600 μM) and RO (37.5–93.75×10^–3^‰) with subsequent 24 h post-cultivation in fresh medium were analyzed similarly as described by Valovicova *et al.* (2009). Briefly, cells were seeded on Petri dishes at a density of 2×10^5^ cells/dish. After treatment, cell cultures were fixed with ice-cold methanol:glacial acetic acid (3:1) for 15 min at room temperature, washed and air dried till next day. Cells thus fixed were stained with DAPI (0.2 μg/ml) diluted in McIlvaine´s buffer (0.2 M Na_2_HPO_4_×2H_2_O, adjusted to pH 7 using citric acid) at room temperature in the dark for 40 min, washed, dried and mounted with glycerol. Cell death (apoptosis and necrosis) was determined using morphological criteria (fragmentation of nuclei) as described by Oberhammer *et al.* (1992) and micronuclei were evaluated as described by Miller *et al.* (1995). Two thousand cells per dish were analyzed using a fluorescence microscope Olympus BX51.

### Electrophoretic analysis of apoptosis

Cells treated with 50–650 μM of CA and 12.5–125×10^–3^‰ of RO for 24 h and after subsequent post-cultivation in fresh medium for 24 and 48 h were harvested, washed in PBS and lysed in 100 μl of lysis solution (10 mM Tris, 10 mM EDTA, 0.5% (w/v) Triton X-100) supplemented with proteinase K (1 mg/ml). Samples were then incubated at 37 °C for 1 h and heated at 70 °C for 10 min. After lysis, RNA-se (200 μg/ml) was added and repeated incubation at 37 °C for 1 h followed. The samples were subjected to electrophoresis at 40 V for 3 h in 1.3% (w/v) agarose gel complemented with EtBr. Separated DNA fragments (DNA ladders) were visualized using a UV transilluminator (302 nm; Jantova *et al.*, 2007).

### Cell cycle analysis

Controls (untreated cells) and HepG2 cells treated with CA at concentrations of 100, 200, 300 and 400 μM or RO at concentrations of 18.75, 37.5, 62.5 and 93.75×10^–3^‰ for 24 h were harvested, washed twice in PBS, centrifuged (10 min, 1500 rpm) and exposed to 0.05% Triton X-100 in PBS supplemented with 1% RNA-se for 20 min at 37°C. Afterwards, DNA was stained by PI (1 mg/ml). Cell cycle was analyzed by a flow cytometer FACS Altra (Beckman-Coulter, USA) with the use of cell cycle analysis software (FACS express 4). A minimum of 10 000 cells per sample were analyzed (Letasiova *et al.*, 2006).

### Statistical analysis

The results are presented as means from at least three sets of independent experiments ± standard deviation (SD). The significance of differences between samples was evaluated by Student´s *t*-test. A *p-*value of <0.05 was considered significant.

## Results

**Figure 1:**
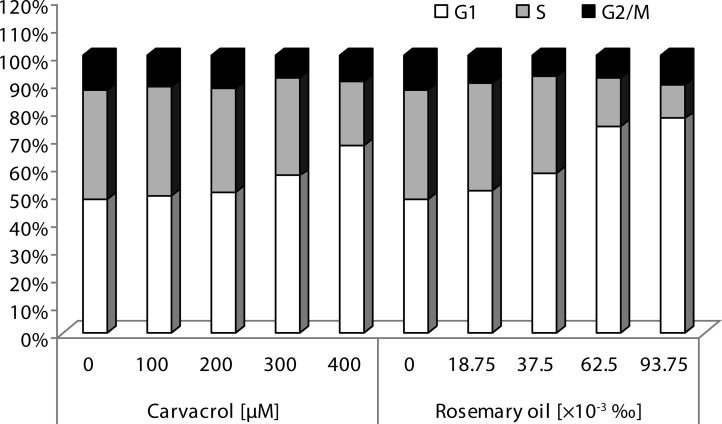
Analysis of cell cycle in HepG2 cells treated with indicated concentrations of carvacrol and rosemary oil for 24 h. Cell cycle phases - G1 (white bars), S (gray bars) and G2/M (black bars) were measured by flow cytometry. A minimum of 10 000 cells per sample were analyzed.

To study the effects of natural volatiles tested on the cell cycle profile we monitored the effect of 24 h treatment with 100, 200, 300 and 400 μM of CA and 18.75, 37.5, 62.5 and 93.75×10^–3^‰ of RO in HepG2 cells (Figure 1). Flow cytometry analysis of cell cycle revealed that CA and RO raised accumulation of cells in the G1 phase gradually in concentration-dependent manner. Simultaneously, reduction in the number of cells in the S phase after 24 h exposure to substances was detected. The cell division was thus slowed down or stopped and this process resulted in cell death.

**Figure 2:**
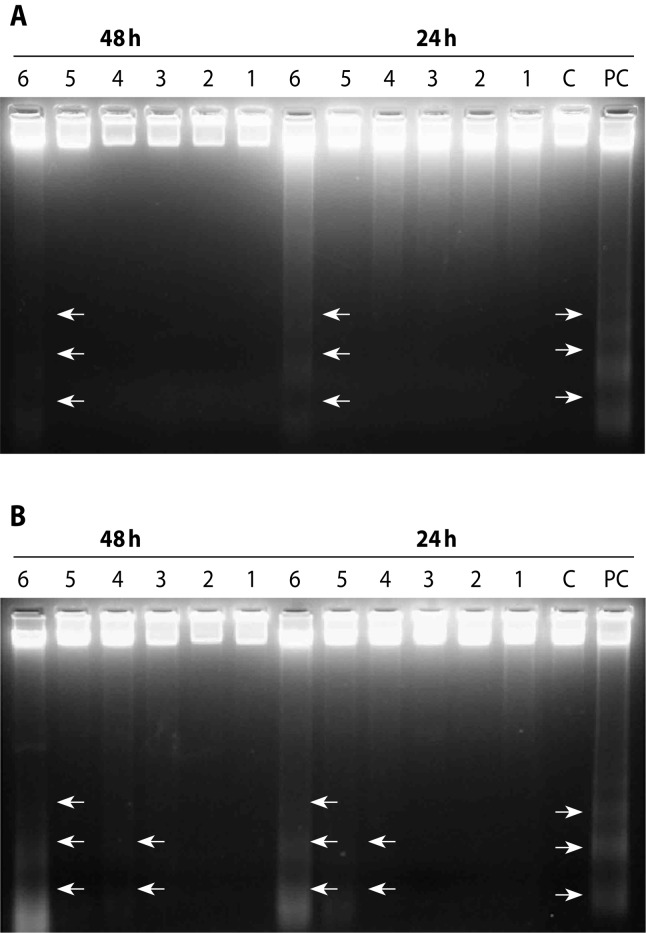
An apoptotic DNA fragmentation induced by carvacrol (A) and rosemary oil (B) in HepG2 cells after 24 and 48 h post-cultivation. C=control cells; PC=positive control; cells treated with 0.5 μM B[a]P for 1 h and then irradiated with UVA light (2.4 J/cm^2^). For carvacrol, the numbers in electrophoretic patterns represent: 1=50 μM; 2=100 μM; 3=200 μM; 4=350 μM; 5=500 μM; 6=650 μM. For rosemary oil, the numbers in electrophoretic patterns represent: 1=12.5×10^–3^‰; 2=18.75×10^–3^‰; 3=37.5×10^–3^‰; 4=62.5×10^–3^‰; 5=93.75×10^–3^‰; 6=125×10^–3^‰.

We investigated the ability of CA and RO to induce apoptosis also by electrophoretic analysis of internucleosomal DNA fragmentation. B[a]P-treated and UVA-irradiated HepG2 cells were used as positive control. HepG2 cells were treated with volatiles for 24 h and subsequently post-cultivated in the medium for 24 and 48 h. As shown in Figure 2A, CA exerted the strongest apoptotic effect at the concentration of 650 μM after 24 h post-cultivation. Figure 2B shows the efficiency of RO to evoke apoptotic cell death in HepG2 cells after 24 and 48 h post-cultivation. A dose-dependent increase in DNA fragmentation was observed. The most intense “DNA ladder” was detected at the highest concentration of rosemary oil (125×10^–3^‰) tested after 24 as well as 48 h of post-cultivation.

**Figure 3:**
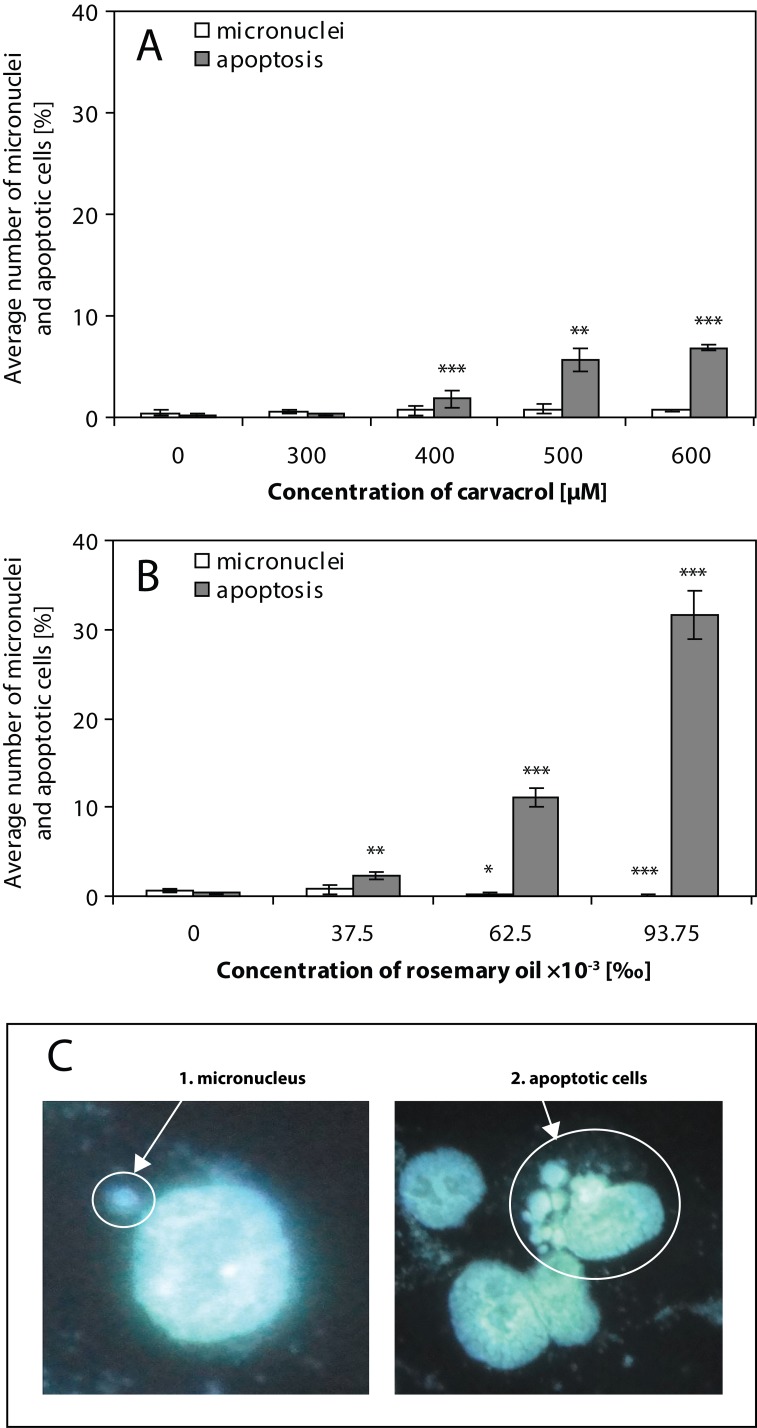
Frequency of micronuclei (white bars; C1) and apoptotic HepG2 cells (gray bars; C2) induced by carvacrol (A) and rosemary oil (B) after 24 h treatment and subsequent 24 h post-cultivation in fresh medium. Percentage of micronuclei and apoptotic HepG2 cells are related to one thousand cells. Values represent the means ± SD. Data were analyzed using Student´s *t*-test; significantly different from the control: *p<0.05; **p<0.01; ***p<0.001.

We simultaneously scanned nuclei of HepG2 cells after 24 h treatment with CA (Figure 3A) and with RO (Figure 3B) and subsequent 24 h post-cultivation for the numbers of both micronuclei and morphological changes typical for apoptosis (Figure 3C). As shown in Figure 3, CA or RO at the concentration ranges tested induced very low, almost no, level of micronuclei (max. 0.67%) together with the rise in the numbers of apoptotic cells. We found that increasing concentrations of both volatiles decreased the rate of cell division (data not shown). Our fluorescent microscopic observations confirmed electrophoretically monitored DNA fragmentation in HepG2 cells after treatment with CA or RO.

## Discussion

Natural products have long been a great source of drugs. However, an advancing development of new anticancer drugs, drug combinations and chemotherapy strategies is still required. There are at least 250 000 species of plants of which more than a third have been found to possess significant anticancer properties (Mukherjee *et al.*, 2001). Plants produce a diverse field of over 100 000 low-molecular-mass natural products, also called secondary metabolites. These metabolites are different from the components of primary metabolism in that they are generally non-essential for the basic metabolic processes of the plant. Most of them are derived from the isoprenoid, phenylpropanoid, alkaloid or fatty acid/polyketide pathways (Dixon, 2001).

Consequently, in our previous experiments we evaluated the DNA-protective potential of CA against damage induced in HepG2 cells by hydrogen peroxide (H_2_O_2_) and *tert*-butyl hydroperoxide, using conventional and modified comet assay (Slamenova *et al.*, 2013b). In our *ex vivo* studies we manifested the ability of CA and RO to protect DNA of hepatocytes and testicular cells isolated from CA- or RO-supplemented rats against several DNA-damaging agents (Slamenova *et al.*, 2008; Horvathova *et al.*, 2010; Slamenova *et al.*, 2011a; Slamenova *et al.*, 2011b). We examined also the anticancer potential of these two volatiles on human hepatoma cell line HepG2 (Melusova *et al.*, 2014a) and on BHNF-1 fibroblasts (Melusova *et al.*, 2014b).

In our previous study we obtained results that showed gradually inhibited cell growth and viability in concentration-dependent manner for both natural volatiles. Growth inhibitory concentrations IC_50_ for 24 h treatment of HepG2 cells were 425 μM for CA and 89×10^–3^‰ for RO. HepG2 cells were more sensitive to both compounds tested in comparison to human fibroblastoid cell line B-HNF-1 for which respective IC_50_ values of 680 μM (CA) and 108×10^–3^‰ (RO) were detected (Melusova *et al.*, 2014b).

Conventional and modified comet assay showed that oxidizing agents H_2_O_2_, DMNQ and cytotoxic concentrations of CA and RO induced DNA damage in HepG2 cells. On the other hand, non-toxic concentrations of CA and RO did not induce the formation of DNA strand breaks, moreover they protected DNA of HepG2 cells against genotoxicity of H_2_O_2_ and DMNQ (Slamenova *et al.*, 2013b; Horvathova *et al.*, 2014; Melusova *et al.*, 2014a).

Our *ex vivo* studies showed that extracts prepared from hepatocytes of CA- and RO-supplemented rats did not increase incision repair activity compared to extracts from liver cells of control animals (Melusova *et al.*, 2014a).

Generally, cytotoxicity of compounds may induce growth inhibition, cell cycle arrest, DNA damage or cell death. Therefore, the aim of this study was to examine the ability of cytotoxic concentrations of CA and RO to induce cell cycle arrest, DNA fragmentation and apoptotic cell death. Flow cytometry was used for cell cycle analysis and to study the ability of volatiles to induce apoptosis in HepG2 cells. In addition, micronucleus assay and electrophoretical detection of internucleosomal DNA fragmentation were used for examination of micronuclei formation and cell death.

Our observations from flow cytometry analysis of the cell cycle showed that CA and RO after 24 h treatment of HepG2 cells increased accumulation of cells in the G1 phase gradually in a concentration-dependent manner.

The ability of natural essential oils to interfere with the cell cycle was found also by other authors. Foo *et al.* (2013) analyzed the cell cycle of leukemic cell lines HL-60, WEHI-3B and K562 treated with essential oils isolated from the seeds of *Hibiscus cannabinus* by flow cytometry. The authors found that the oils tested caused cell cycle arrest in the G1 phase and significantly increased the apoptotic population of leukemic cells.

Liu *et al.* (2013) investigated the effect of germacrone, one of the main bioactive components of *Rhizoma curcuma*, on the cell cycle of HepG2 cells after 24 h exposure. An accumulation of cells in the G2/M phase coupled with a decreasing number of cells in the G1 phase was observed at the highest concentration of germacrone (200 μM).

DNA damage plays an important role in the processes of mammalian cells death. Reactions involving oxidative stress and ROS production, resulting in chromatin damage in cells such as DNA fragmentation, lead to cell death by apoptosis or necrosis. Some substances induce a formation of high molecular breaks (50 kbp – 1 Mbp) and internucleosomal DNA fragments in different types of mammalian cells during cell death (Higuchi, 2003). Apoptosis and necrosis are two distinct forms of cell death which have quite different effects on the surrounding tissues. In addition to conventional morphological and biochemical characteristics, apoptosis is characterized by DNA fragmentation in internucleosomal joints, leading to the creation of separate strips – “bends” in multiples of 180–200 bp and the formation of „DNA ladder“ (Carson & Ribeiro, 1993). Various papers and our previous experiments (Melusova *et al.*, 2014a) showed that some natural substances at high concentrations act as prooxidants which induce an increased production of ROS and cause DNA damage that may lead to cell death.

In our study we investigated the ability of CA and RO to induce apoptotic cell death in HepG2 cells after 24 h treatment using three methods. Flow cytometry showed that increasing concentrations of CA and RO decreased the number of viable cells in the samples. At the same time, an increased percentage of cells was observed in the late stage of apoptosis (data not shown). Internucleosomal DNA fragmentation and formation of apoptotic bodies was clearly detected at the highest concentrations of both natural volatiles tested. In addition, the micronucleus test showed that CA and RO induced characteristic apoptotic morphological changes in HepG2 cells. On the other hand, neither of the oils tested did display clastogenic activity – almost no micronuclei were formed in comparison to the control cells. Necrotic cells were not observed.

Based on the results obtained from all three methods used we can conclude that flow cytometry and agarose gel electrophoresis showed that CA and RO induced apoptotic cell death of HepG2 cells. Microscopic observations were in accordance with these results.

The induction of apoptotic death of cells treated with essential oils and carvacrol were observed by several authors. Arunasrre (2010) monitored the influence of carvacrol on the occurrence of apoptosis in human metastatic breast MDA-MB231 cells using analysis of the cell cycle and Annexin V staining. The results showed that carvacrol clearly induced apoptosis in the cells, as reflected in the release of mitochondrial cytochrome c, activation of caspases and cleavage of PARP protein. The findings also revealed that rising concentrations of carvacrol increased the number of apoptotic cells in the G0/G1 phase while the incidence of cells in the S phase was reduced. Thus carvacrol induced apoptosis in tumor cells and inhibited DNA synthesis in the S phase of the cell cycle.

Induction of apoptotic cell death caused by some plants was observed by Kontogianni *et al.* (2013). The ability of an extract isolated from the leaves of *Rosmarinus officinalis* to induce internucleosomal DNA cleavage in rat insulinoma RINm5F cells was investigated. The authors observed an incidence of DNA fragments released into the cytoplasm in the form of DNA-histone complexes and their presence in apoptotic cells was detected. Rosemary extract caused apoptosis depending on the concentration.

Essential oils extracted from *Origanum onites* L. and carvacrol caused morphological changes in HepG2 cells after 48 h, such as chromatin condensation and shrinkage of the cytoplasmic membrane (Sivas & Tomsuk, 2011).

Wu *et al.* (2012) investigated the effect of ganoderic acid DM, isolated from a plant of *Ganoderma lucidum*, on the form of cell death induced in human breast cancer MCF-7 cells. They found a clear appearance of DNA fragments detected by gel electrophoresis. Cell death was also confirmed by cleavage of PARP protein, which is a marker of apoptosis. The constituent of *Ganoderma lucidum* also caused cell cycle arrest in the G1 phase, while the S phase had a decreasing tendency.

On the basis of the presented results we can conclude that while cytotoxic concentrations of CA and RO caused DNA damage in HepG2 cells, they did not display clastogenic activity in the micronucleus test. Flow cytometry indicated that damaged cells were blocked in the G1 phase of the cell cycle and signaling pathways leading to cell death by apoptosis were probably activated.
